# Outcomes of Vitrectomy with 50% Fluid–Air Exchange for Idiopathic Macular Hole

**DOI:** 10.3390/jcm15145606

**Published:** 2026-07-17

**Authors:** Rinako Miki, Manabu Yamamoto, Satoshi Honda, Shigeru Honda

**Affiliations:** 1 Department of Ophthalmology and Visual Science, Graduate School of Medicine, Osaka Metropolitan University, Osaka 545-8585, Japan; zk.rinako@gmail.com (R.M.); hondaskywalkerc3po@gmail.com (S.H.); shonda@omu.ac.jp (S.H.); 2 Kashiwara Municipal Hospital, 1-7-9 Hozenji, Kashiwara, Osaka 582-0005, Japan

**Keywords:** idiopathic macular hole, vitrectomy, fluid–air exchange, air tamponade, optical coherence tomography, face-down positioning

## Abstract

**Background/Objectives**: Vitrectomy with gas tamponade is the standard treatment for idiopathic macular hole (MH); however, prolonged facedown positioning (FD) and delayed postoperative assessment of MH closure may increase patient burden. This study aimed to evaluate the anatomical and functional outcomes of vitrectomy with 50% fluid–air exchange for idiopathic MH, with particular emphasis on the feasibility of early postoperative OCT assessment and individualized postoperative management. **Methods**: This retrospective study included 22 eyes from 19 patients who underwent 27-gauge pars plana vitrectomy with internal limiting membrane peeling and 50% fluid–air exchange for idiopathic MH between April 2019 and January 2025. MH closure was evaluated postoperatively using spectral-domain optical coherence tomography (OCT), and FD was discontinued once closure was confirmed. The primary outcome was the MH closure rate. Secondary outcomes included time to closure, changes in best-corrected visual acuity (BCVA), and complications. **Results**: Primary MH closure was achieved in 20 of 22 eyes (90.9%). The mean time to closure was 1.7 days, and closure was confirmed on postoperative day 1 in 14 eyes (64%). MH status was successfully evaluated using conventional SD-OCT in all 22 eyes (100%), and no eye required swept-source OCT imaging. Two eyes required reoperation with complete fluid–air exchange, after which both achieved closure. Mean BCVA improved significantly from 0.45 ± 0.30 logMAR preoperatively to 0.15 ± 0.31 logMAR postoperatively (*p* < 0.001). No severe intraoperative or postoperative complications, including retinal detachment or endophthalmitis, were observed. **Conclusions**: Vitrectomy with 50% fluid–air exchange enabled early postoperative assessment of MH status using conventional SD-OCT and facilitated individualized management of facedown positioning. Further prospective comparative studies are warranted to define its role in contemporary MH surgery.

## 1. Introduction

Idiopathic macular hole (MH) is a full-thickness defect of the neurosensory retina at the fovea and is generally considered to develop secondary to vitreomacular traction [1,2]. Since the introduction of pars plana vitrectomy for MH treatment, anatomical and functional outcomes have improved dramatically owing to advances in vitreoretinal surgical techniques. In particular, the introduction of internal limiting membrane (ILM) peeling and gas tamponade has substantially increased closure rates, with many studies reporting primary anatomical success rates exceeding 90% [3].

Despite these favorable surgical outcomes, postoperative management following MH surgery remains an important clinical issue. Facedown positioning (FD) is commonly recommended after surgery to facilitate apposition of the hole edges and contact between the tamponade agent and the macula. However, prolonged FD may impose considerable physical and psychological burden on patients, including neck and back pain, sleep disturbance, and reduced quality of life [4,5,6,7,8]. Recent studies have questioned the necessity of prolonged FD in selected cases, suggesting that postoperative management may be individualized rather than uniformly prescribed [6,7,8].

Several factors have been reported to influence MH closure and postoperative visual outcomes, including minimum hole diameter, symptom duration, MH stage, and surgical technique [9,10,11]. Although modern vitreoretinal surgery achieves high closure rates in many patients, the optimal strategy for postoperative management remains less clearly defined. Recent studies have questioned the necessity of prolonged FD in selected MH cases and have suggested that postoperative management may be individualized according to anatomical findings rather than predetermined postoperative protocols [6,7,8,12].

Another important limitation of conventional MH surgery is the difficulty of evaluating macular hole status during the early postoperative period. Because the vitreous cavity is almost completely filled with gas, optical coherence tomography (OCT) imaging is often difficult immediately after surgery [12,13]. As a result, confirmation of MH closure may be delayed, and FD duration is frequently determined empirically rather than based on direct anatomical assessment. This limitation may result in unnecessary prolongation of postoperative restrictions in some patients.

Partial fluid–air exchange may provide a practical solution to these issues. By intentionally preserving a portion of intraocular fluid, postoperative visualization of the macula may become feasible using conventional OCT equipment during the early postoperative period [12,13,14]. Such an approach could support OCT-guided postoperative management, individualized FD duration, earlier discharge, and timely reoperation when closure is not achieved. Importantly, the purpose of this strategy is not necessarily to maximize anatomical closure rates through more intensive tamponade techniques, but rather to optimize postoperative management based on actual anatomical findings.

Clinical evidence regarding vitrectomy with partial fluid–air exchange for idiopathic MH remains limited. Therefore, the present study aimed to evaluate the anatomical and functional outcomes of vitrectomy with 50% fluid–air exchange, with particular emphasis on the feasibility of early postoperative OCT assessment and individualized postoperative management.

## 2. Materials and Methods

### 2.1. Study Design and Ethics

This retrospective observational study was conducted at Osaka Metropolitan University Hospital. The study adhered to the tenets of the Declaration of Helsinki and was approved by the Institutional Review Board of Osaka Metropolitan University Graduate School of Medicine (protocol code 2019-062; date of approval: 16 December 2019). Written informed consent was obtained from all subjects involved in the study.

### 2.2. Study Participants

We retrospectively reviewed consecutive patients who underwent vitrectomy for idiopathic MH with 50% fluid–air exchange between April 2019 and January 2025 at Osaka Metropolitan University Hospital. Eyes with high myopia, macular hole retinal detachment, peripheral retinal breaks, or other retinal diseases that could affect surgical outcomes were excluded. Eyes with insufficient postoperative follow-up were also excluded from the analysis. Patient demographics and baseline clinical characteristics, including age, sex, axial length, preoperative best-corrected visual acuity (BCVA), minimum linear diameter of the MH, and Gass classification stage, were reviewed from the medical records.

### 2.3. Surgical Procedure

**Figure 1:**
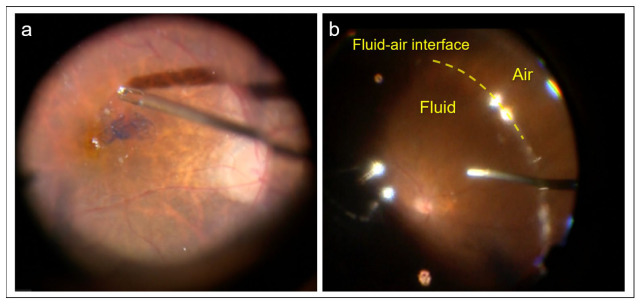
Surgical procedure of vitrectomy with 50% fluid–air exchange for idiopathic macular hole. (**a**) Internal limiting membrane peeling was performed around the macular hole after staining. (**b**) Fluid–air exchange was intentionally terminated when the fluid–air interface reached approximately the equatorial level, resulting in an estimated intraocular air fill of approximately 50%.

All surgeries were performed by a single experienced vitreoretinal surgeon using a 27-gauge pars plana vitrectomy system. Combined phacoemulsification and intraocular lens implantation were performed simultaneously in phakic eyes, whereas one eye had previously undergone cataract surgery and was pseudophakic at the time of MH surgery. After core vitrectomy, posterior vitreous detachment was confirmed and additional peripheral vitrectomy was performed as needed to relieve vitreoretinal traction. Care was taken to minimize residual cortical vitreous around the macular region before ILM peeling. ILM peeling was performed in all eyes using indocyanine green or brilliant blue G staining to facilitate visualization of the ILM (Figure 1a). The peeled area included the foveal region surrounding the MH. Fluid–air exchange was subsequently performed. Air infusion was continued until the fluid–air interface reached approximately the equatorial region of the eye, resulting in an estimated intraocular air fill of approximately 50% (Figure 1b). The surgeon visually confirmed the fluid–air interface level intraoperatively, and the exchange procedure was intentionally terminated before complete air filling of the vitreous cavity. This approach was intended to preserve partial intraocular fluid while maintaining sufficient tamponade effect around the macular region. No expansile gas tamponade was used during the initial surgery.

### 2.4. Postoperative Assessment and Facedown Positioning

Postoperative MH closure was evaluated using spectral-domain OCT (Spectralis^®^, Heidelberg Engineering GmbH, Heidelberg, Germany) beginning on postoperative day 1. Swept-source OCT (DRI OCT Triton^®^, Topcon Corporation, Tokyo, Japan) was available as an alternative imaging modality when SD-OCT visualization was considered insufficient. However, closure status could be evaluated using SD-OCT alone in all eyes included in the present study, and no eye required SS-OCT imaging for determination of MH closure. The postoperative day on which definite anatomical closure of the MH was confirmed was defined as the closure day. Patients were instructed to maintain FD immediately after surgery until MH closure was confirmed by OCT examination. FD was discontinued immediately after anatomical closure was confirmed. Postoperative examinations were generally performed daily during the early postoperative period until definitive MH closure was identified. At our institution, patients undergoing MH surgery are routinely hospitalized during the immediate postoperative period. Therefore, serial OCT examinations were incorporated into standard inpatient postoperative care and did not require additional outpatient visits. Closure confirmation was also used to guide discharge planning. All patients received standard postoperative topical antibiotic and corticosteroid eye drops. When postoperative OCT demonstrated persistent MH opening without a tendency toward closure during the early postoperative course, additional surgical intervention was considered. Reoperation with complete fluid–air exchange was performed at the surgeon’s discretion based on postoperative OCT findings.

### 2.5. Outcome Measures

The primary outcome measure was the anatomical closure rate of the MH after the initial surgery. The feasibility of early postoperative OCT assessment was also evaluated by determining whether MH status could be assessed using conventional SD-OCT during the early postoperative period. Secondary outcome measures included time to MH closure, changes in BCVA, and intraoperative or postoperative complications. BCVA measurements were converted to the logarithm of the minimum angle of resolution (logMAR) for statistical analysis. Postoperative BCVA was evaluated at the final follow-up visit.

### 2.6. Statistical Analysis

Descriptive statistics were used to summarize baseline characteristics and surgical outcomes. Continuous variables are presented as mean ± standard deviation, and categorical variables are presented as numbers and percentages. Preoperative and postoperative BCVA values were compared using the Wilcoxon signed-rank test. Because the primary purpose of this study was descriptive evaluation of surgical outcomes and postoperative management, no formal subgroup analysis was performed. Three patients contributed both eyes to the study cohort. Statistical significance was defined as a *p*-value < 0.05. Statistical analyses were performed using IBM SPSS Statistics for Windows, version24.0 (IBM Corp., Armonk, NY, USA).

## 3. Results

### 3.1. Patient Characteristics

A total of 22 eyes from 19 patients were included in this study. Baseline patient characteristics are summarized in Table 1, and individual patient characteristics and postoperative outcomes are provided in Supplementary Table S1. The mean patient age was 66.1 ± 7.7 years (range, 54–80 years). Seven eyes (31.8%) were from male patients and 15 eyes (68.2%) were from female patients. One eye (4.5%) was pseudophakic at the time of surgery. The mean axial length was 24.66 ± 1.46 mm. The mean preoperative BCVA was 0.45 ± 0.30 logMAR. The mean minimum linear diameter of the MH was 311.4 ± 191.4 µm. The mean basal diameter (BD) was 731.0 ± 333.0 μm. According to the Gass classification, nine eyes (40.9%) were classified as stage II, five eyes (22.7%) as stage III, and eight eyes (36.4%) as stage IV. Three patients contributed both eyes to the study cohort (Table 1).

### 3.2. Early Postoperative OCT Assessment

**Figure 2:**
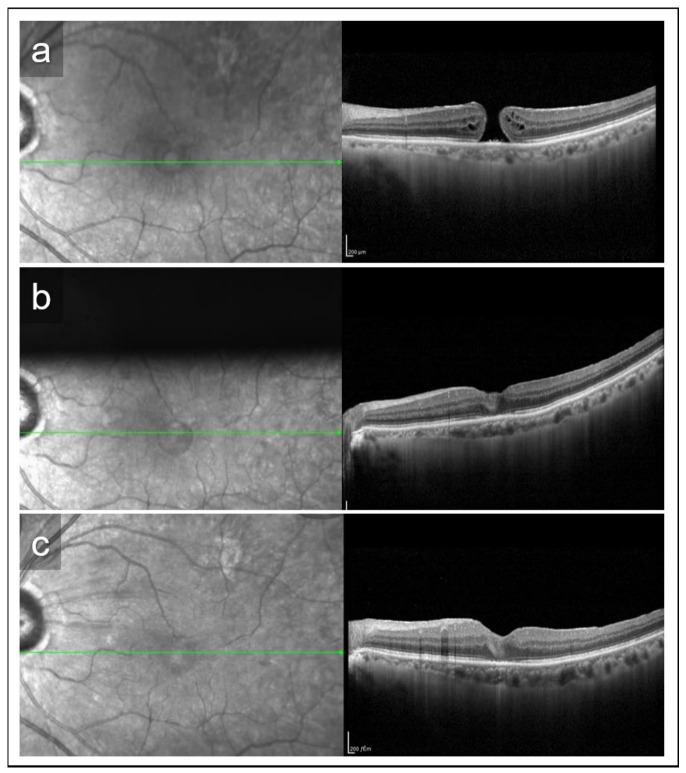
Representative case with successful primary macular hole closure after vitrectomy with 50% fluid–air exchange. A 74-year-old man with stage IV idiopathic macular hole underwent combined cataract surgery and vitrectomy with internal limiting membrane peeling and 50% fluid–air exchange. The green arrows indicate the position of the OCT B-scan. (**a**) Preoperative OCT image demonstrating a full-thickness macular hole. (**b**) OCT image obtained on postoperative day 1 showing successful anatomical closure of the macular hole. (**c**) OCT image obtained 1 month after surgery demonstrating restoration of the foveal contour. Best-corrected visual acuity improved from 0.6 preoperatively to 0.7 postoperatively.

**Figure 3:**
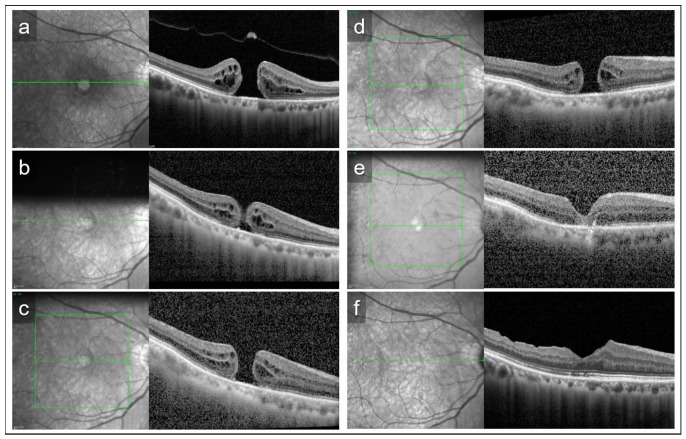
Representative case requiring reoperation after persistent macular hole following vitrectomy with 50% fluid–air exchange. A 66-year-old woman with stage III idiopathic macular hole underwent combined cataract surgery and vitrectomy with internal limiting membrane peeling and 50% fluid–air exchange. The green arrows indicate the position of the OCT B-scan. (**a**) Preoperative OCT image demonstrating a full-thickness macular hole. (**b**–**d**) OCT images obtained on postoperative days 1, 2, and 3 showing persistent non-closure of the macular hole. (**e**) OCT image obtained 5 days after reoperation with complete fluid–air exchange demonstrating successful anatomical closure. (**f**) OCT image obtained 1 year after reoperation showing restoration of the foveal contour. Best-corrected visual acuity improved from 0.3 preoperatively to 0.7 at 12 months postoperatively.

Early postoperative assessment of MH status was successfully performed using conventional spectral-domain OCT in all 22 eyes (100%). No eye required additional swept-source OCT imaging to determine closure status. Representative postoperative OCT images are shown in Figure 2 and Figure 3. MH status could be evaluated beginning on postoperative day 1, allowing serial anatomical assessment during the early postoperative period.

### 3.3. Anatomical Outcomes

**Figure 4:**
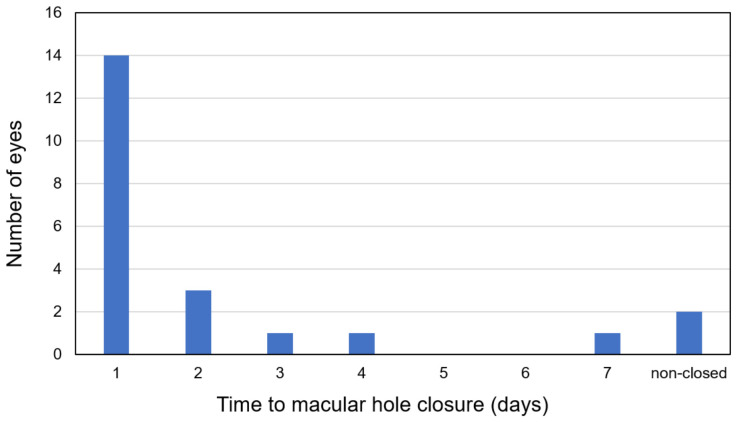
Distribution of time to macular hole closure after surgery. Most eyes achieved anatomical closure within the early postoperative period, with closure confirmed on postoperative day 1 in 14 eyes. Two eyes did not achieve primary closure after the initial surgery.

Representative OCT images of both successful primary closure and persistent MH requiring reoperation are shown in Figure 2 and Figure 3. Primary MH closure was achieved in 20 of 22 eyes, corresponding to a primary closure rate of 90.9%. The mean time to closure was 1.7 days (range, 1–7 days). Among the 20 eyes with successful primary closure, closure was confirmed on postoperative day 1 in 14 eyes (64%). Closure was confirmed within postoperative day 3 in 18 of 20 successfully closed eyes (90%). Closure timing is summarized in Figure 4. One eye achieved closure on postoperative day 7 despite progressive reduction in the intraocular air volume. This eye had a BD of 746 μm and an MLD of 239 μm. Two eyes did not achieve primary closure after the initial surgery. In both cases, persistent MH was identified during the early postoperative period using OCT imaging. Reoperation with complete fluid–air exchange was subsequently performed within 3–4 days after the initial surgery, and anatomical closure was ultimately achieved in both eyes. One of the reoperation cases had the largest MH in the cohort (BD 1436 μm, MLD 646 μm). In contrast, the other reoperation case had a BD of 723 μm and an MLD of 249 μm, values comparable to those observed in many successfully closed eyes. Notably, the delayed-closure case (closure on postoperative day 7) had a similar BD (746 μm) and MLD (239 μm) to the latter reoperation case.

### 3.4. Visual Outcomes

**Figure 5:**
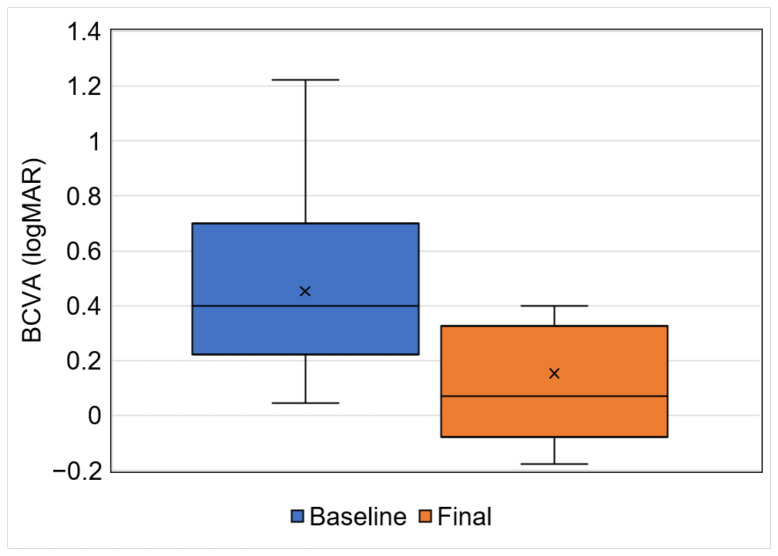
Box-and-whisker plots showing preoperative and postoperative BCVA values expressed in logarithm of the minimum angle of resolution (logMAR). Postoperative BCVA improved significantly compared with preoperative BCVA (*p* < 0.001, Wilcoxon signed-rank test). Boxes indicate the interquartile range, horizontal lines indicate the median, whiskers indicate the range, and × indicates the mean value.

Postoperative BCVA improved significantly compared with preoperative BCVA. The mean BCVA improved from 0.45 ± 0.30 logMAR preoperatively to 0.15 ± 0.31 logMAR postoperatively during a mean follow-up period of 6.2 ± 5.0 months. This improvement was statistically significant (*p* < 0.001, Wilcoxon signed-rank test) (Figure 5).

### 3.5. Complications

No severe intraoperative or postoperative complications associated with significant visual loss, such as endophthalmitis or retinal detachment, were observed during the follow-up period. No retinal detachment, endophthalmitis, or clinically significant intraocular pressure elevation requiring additional treatment occurred during the study period. No patient required unplanned postoperative intervention other than the two eyes that underwent reoperation because of persistent MH.

## 4. Discussion

Previous studies have also explored reduced or absent tamponade strategies for MH surgery, suggesting that successful closure may be achieved even with less intensive postoperative tamponade in selected cases [15]. The principal objective of the present study was not to maximize anatomical closure rates, but to evaluate whether partial fluid–air exchange could facilitate early postoperative assessment of MH status and support individualized postoperative management. Previous studies have reported favorable anatomical outcomes using air tamponade instead of expansile gas for idiopathic MH repair, particularly in small- to medium-sized MHs [16,17]. In the present study, MH status could be evaluated with conventional SD-OCT in all eyes during the early postoperative period, allowing prompt discontinuation of facedown positioning after closure confirmation and timely identification of persistent non-closure requiring additional intervention.

While long-acting gas tamponade and advanced techniques such as the inverted ILM flap may achieve higher closure rates in selected challenging cases, they are not necessarily required for all MHs. The present strategy was developed from a different perspective. Rather than maximizing tamponade duration, it was designed to facilitate postoperative decision-making based on direct anatomical assessment. One of the most important findings of the present study was that postoperative MH status could be evaluated using conventional SD-OCT in all eyes. Previous studies have demonstrated that swept-source OCT can visualize MH morphology in gas-filled eyes [12,13,14]. However, in the present series, no eye required SS-OCT for determination of closure status. Therefore, the potential advantage of partial fluid–air exchange may not be superior image quality itself, but rather the ability to perform reliable early postoperative assessment using standard OCT equipment commonly available in clinical practice.

Early confirmation of MH closure allowed individualized discontinuation of facedown positioning [6,7,8,12,18]. Because patients in our institution routinely remain hospitalized during the immediate postoperative period, serial OCT examinations were incorporated into standard inpatient postoperative care and did not require additional outpatient visits. Closure confirmation also facilitated discharge planning. Therefore, the clinical value of this strategy may be particularly relevant in settings where postoperative management is performed during hospitalization.

Interestingly, one eye achieved closure on postoperative day 7 despite progressive reduction in the intraocular air volume. This eye had a basal diameter of 746 μm and a minimum linear diameter of 239 μm, values comparable to those of many eyes that achieved earlier closure. This observation suggests that successful closure may not depend solely on continuous mechanical tamponade. Biological processes, including gradual approximation of the hole edges after ILM peeling, may continue to contribute to closure during the early postoperative period [14]. These findings support the concept that postoperative management should be guided by actual anatomical findings rather than a predetermined duration of facedown positioning.

Another notable finding was the ability to identify persistent non-closure shortly after surgery. Two eyes required reoperation because OCT demonstrated persistent MH opening during the early postoperative period. Both eyes subsequently achieved anatomical closure after complete fluid–air exchange. One of these eyes had the largest MH in the cohort (BD 1436 μm, MLD 646 μm), suggesting that caution may be warranted when applying this strategy to very large MHs [19].

In contrast, the second reoperation case had a BD of 723 μm and an MLD of 249 μm, values similar to those observed in the delayed-closure case and in many successfully closed eyes. These findings suggest that MH size alone may not fully explain postoperative closure behavior [19,20]. Additional anatomical or biological factors not captured by conventional preoperative measurements may influence closure dynamics. Therefore, early postoperative OCT assessment may be valuable because closure status cannot always be predicted solely from preoperative hole size.

The present findings should not be interpreted as evidence that 50% fluid–air exchange is superior to conventional gas tamponade techniques. Because this study lacked a control group, direct comparison of closure rates was not possible. Rather, the present results suggest that partial fluid–air exchange may represent a management-oriented strategy that enables individualized FD duration, early identification of treatment failure, and prompt reintervention when necessary.

Several limitations should be acknowledged. First, this was a retrospective study conducted at a single institution with a relatively small sample size. Second, there was no direct comparison with conventional air or gas tamponade techniques, precluding definitive conclusions regarding relative efficacy. Third, three patients contributed both eyes to the study cohort. Fourth, postoperative quality of life, patient satisfaction, and speed of return to daily activities were not quantitatively assessed. Finally, the applicability of this strategy to very large MHs remains uncertain and requires further investigation in larger prospective studies.

Nevertheless, the present study demonstrated that 50% fluid–air exchange enabled early SD-OCT-based assessment of MH status in all eyes and supported individualized postoperative management. These findings provide proof-of-concept that postoperative management strategies may be tailored according to actual anatomical findings rather than predetermined positioning schedules alone.

## 5. Conclusions

In conclusion, vitrectomy with 50% fluid–air exchange enabled early postoperative assessment of macular hole status using conventional spectral-domain OCT and supported individualized postoperative management. Closure status could be evaluated in all eyes during the early postoperative period, allowing tailored discontinuation of facedown positioning and prompt identification of persistent non-closure requiring additional intervention.

Rather than maximizing anatomical success through more intensive tamponade strategies, this approach may provide a practical means of optimizing postoperative management based on actual anatomical findings. In our inpatient setting, early confirmation of closure also facilitated discharge planning and reduced unnecessary continuation of facedown positioning.

Although the present study was not designed to compare closure rates with conventional tamponade techniques, the findings provide proof-of-concept that partial fluid–air exchange may serve as a management-oriented strategy for selected cases of idiopathic macular hole. Further prospective comparative studies are warranted to determine its role in contemporary macular hole surgery.

## Figures and Tables

**Table 1:** Baseline characteristics.

Characteristics	Mean ± SD or NO. (%)
No, eyes	22
Age, years	66.1 ± 7.7
Gender (male), eyes	7 (31.8)
Intraocular lens, eyes	1 (4.5)
Axial length, mm	24.66 ± 1.46
Baseline BCVA, logMAR	0.45 ± 0.30
Minimum linear diameter, µm	311.4 ± 191.4
Gass classification	
Stage II	9 (40.9)
Stage III	5 (22.7)
Stage IV	8 (36.4)

## Data Availability

The data presented in this study are available in the Supplementary Materials.

## Supplementary Materials

The following supporting information can be downloaded at https://www.mdpi.com/article/10.3390/jcm15145606/s1. Table S1. Individual patient characteristics and postoperative outcomes.
