# Supplementary material for: Outcomes of Vitrectomy with 50% Fluid–Air Exchange for Idiopathic Macular Hole
Source: J Clin Med. 2026 Jul 17;15(14):5606. doi: 10.3390/jcm15145606 (PMC13412832; doi:10.3390/jcm15145606)
Supplement: Supplementary file 1 [file jcm-15-05606-s001.zip › jcm-4340839-supplementary.pdf]

Table S1. Individual patient characteristics and postoperative outcomes.

No	Sex	RL	Operation	Age	Axial ler	GASS	BD	MLD	close day	Baselin	1M	3M	6M	12M	Duratior	logMAR	1M	3M	6M	12M	Baseline	Max VA
1	M	R	2019/4/1	63	24.81	2	669	277	1	0.2	0.4	0.3	0.7	0.7	12	0.70	0.40	0.52	0.15	0.15	0.70	0.15
2	F	R	2019/4/4	57	25.51	3	334	231	1	0.6	0.8	0.9	0.9	1	12	0.22	0.10	0.05	0.05	0.00	0.22	0.00
	F	L	2021/6/21	59	25.3	2	671	374	2	0.5	0.9	-	-	-	1	0.30	0.05				0.30	0.05
3	F	R	2019/4/11	68	23.01	2	341	203	1	0.7	0.9	0.9	1	1.5	12	0.15	0.05	0.05	0.00	-0.18	0.15	-0.18
4	F	R	2019/5/20	66	22.16	4	723	249	-	0.8	1	1	1	1	12	0.10	0.00	0.00	0.00	0.00	0.10	0.00
5	M	L	2019/6/27	74	25.51	2	316	171	4	0.3	1	1	0.9	1	12	0.52	0.00	0.00	0.05	0.00	0.52	0.00
6	F	R	2020/2/17	66	23.45	3	1436	646	-	0.3	0.3	0.4	0.4	0.7	12	0.52	0.52	0.40	0.40	0.15	0.52	0.15
7	F	R	2020/3/30	71	23.32	4	139	82	1	0.9	1	1.5	1.2	1.2	12	0.05	0.00	-0.18	-0.08	-0.08	0.05	-0.18
8	F	L	2020/6/5	54	25.88	4	487	124	2	0.2	0.9	-	-	1.2	12	0.70	0.05			-0.08	0.70	-0.08
	F	R	2025/1/10	58	26.42	4	422	56	1	0.9	1	1.2	-	-	3	0.05	0.00	-0.08			0.05	-0.08
9	F	L	2020/8/13	77	25.06	2	1272	354	1	0.06	0.05	-	-	-	1	1.22	1.30				1.22	1.30
10	F	L	2020/10/26	60	24.4	3	932	736	1	0.4	0.4	-	-	-	1	0.40	0.40				0.40	0.40
11	M	R	2021/5/14	70	23.56	2	597	50	3	0.15	0.9	0.9	-	1.2	12	0.82	0.05	0.05		-0.08	0.82	-0.08
	M	L	2024/11/29	74	23.36	2	692	224	1	0.3	0.4	-	-	-	1	0.52	0.40				0.52	0.40
12	F	R	2021/8/6	80	24.93	3	788	201	1	0.6	0.6	-	-	-	1	0.22	0.22				0.22	0.22
13	M	R	2021/10/8	54	28.6	3	746	239	7	0.6	0.8	1	1.2	-	6	0.22	0.10	0.00	-0.08		0.22	-0.08
14	F	L	2021/12/10	69	23.53	2	921	703	1	0.1	0.4	-	-	-	1	1.00	0.40				1.00	0.40
15	F	L	2022/2/7	58	26.86	4	1119	529	1	0.2	0.3	0.8	0.8	-	6	0.70	0.52	0.10	0.10		0.70	0.10
16	F	R	2022/2/21	66	23.1	2	576	278	1	0.4	1	-	-	-	1	0.40	0.00				0.40	0.00
17	F	R	2022/4/11	60	24.59	4	788	428	1	0.3	0.5	0.15	-	-	3	0.52	0.30	0.82			0.52	0.30
18	M	L	2022/4/11	74	25.16	4	890	358	1	0.6	0.7	-	-	-	1	0.22	0.15				0.22	0.15
19	M	R	2025/1/31	77	23.92	4	1222	338	2	0.4	0.4	0.4	-	-	3	0.40	0.40	0.40			0.40	0.40
